# Onychodystrophy with Multiple Epiphyseal Dysplasia: Literature Review

**DOI:** 10.1159/000528474

**Published:** 2023-01-19

**Authors:** Samantha J. Albucker, Shari R. Lipner

**Affiliations:** ^a^Tulane University School of Medicine, New Orleans, Louisiana, USA; ^b^Department of Dermatology, Weill Cornell Medicine, New York, New York, USA

**Keywords:** Onychodystrophy, Nail dystrophy, Multiple epiphyseal dysplasia

## Abstract

**Introduction:**

Onychodystrophy has been described in association with certain bone disorders, including Nail-Patella Syndrome, Hutchinson-Gilford Progeria Syndrome, Coffin-Siris Syndrome, and congenital brachydactyly. However, nail changes associated with multiple epiphyseal dysplasia (MED) has not been documented.

**Case Presentation:**

An 11-year-old male with history of MED presented with thickened, dystrophic appearing fingernails. Physical examination was significant for fingernail longitudinal ridges and grooves, thinning, and distal splitting. Dermoscopy revealed superficial desquamation. Nail clippings were negative for microbial pathogens. Hand X-rays showed brachydactyly, shortening of the metacarpals, and sclerotic epiphyses of the bilateral 5th distal phalanges and right 2nd distal phalanx.

**Conclusion:**

This is the first documented case of MED with onychodystrophy, supporting the link between phalangeal formation and nail development. It is important to perform a careful examination of the nail units in patients with skeletal dysplasia and screen patients with characteristic and unexplained nail changes for bony changes. Living with skeletal disease is extremely challenging, and treatment of associated nail disease can improve quality of life for these patients.

## Established Facts

- Multiple epiphyseal dysplasia is an autosomal dominant chondrodysplasia that presents with mild-to-moderate short stature, and osteoarthritis mainly of the knee and hip joints.
- Nail changes have been associated with skeletal dysplasia disorders, including brachydactyly and nail hypoplasia, Nail-Patella Syndrome, Hutchinson-Gilford Progeria Syndrome, and Coffin-Siris Syndrome.

## Novel Insights

- We present a case of multiple epiphyseal dysplasia with onychodystrophy, reinforcing the connection between nail changes and skeletal deformities.

## Introduction

Onychodystrophy has been described in association with certain bone disorders, including Nail-Patella syndrome, Hutchinson-Gilford Progeria syndrome, Coffin-Siris syndrome, and congenital brachydactyly, demonstrating nail formation is in part bone dependent.

Multiple epiphyseal dysplasia (MED) is an autosomal dominant skeletal dysplasia, affecting 1 in 10,000 people [1, 2, 3, 4]. It commonly presents with osteoarthritis before the age of 30, usually in large weight-bearing joints such as the hip or the knee [1, 2]. It is thought to be due to delayed and irregular ossification of the epiphyses [1]. Mutations in five genes have been identified, including COMP (EDM1), COL9A1, COL9A2, COL9A3, and MATN3, making it one of the most genetically heterogeneous disorders [1]. COMP, encoding cartilage oligomeric matrix protein, is mutated in 80% of cases [1]. COL9A1, COL9A2, and COL9A3 encode for 3 alpha chains of cartilage-specific type IX collagen [1, 3, 4]. MATN3 encodes for matrillin-3, which is a cartilage extracellular matrix protein [1, 3, 4]. MED is diagnosed based on clinical presentation and radiological imaging [3]. Onychodystrophy in patients with MED has not previously been described. Herein, we present a case of onychodystrophy in a child with a history of MED.

## Case Report

An 11-year-old male presented with thickened, dystrophic appearing fingernails for 1 year. He felt embarrassed at school and often wore bandages to cover his nails. He had no difficulty picking up coins or performing activities of daily life. He denied scalp involvement, skin rash, or picking or biting the nails.

There was no personal or family history of skin disorders. He had seen an outside dermatologist who performed a nail clipping that was negative for a fungal infection. He had used ketoconazole cream and lac-hydrin cream under occlusion for several months each with no improvement.

As an infant, he had difficulty bending one of his legs while crawling and had a persistently abnormal gait. He was evaluated by orthopedics who felt his physical findings were demonstrative of MED. Hip and pelvis X-rays showed small, irregular epiphyseal and apophyseal ossifications centers, representative of MED. His father had genetic testing that identified a mutation in COMP c.1021_1026delGAGGAC, p.Glu341_Asp342del2, consistent with a diagnosis of COMP-related MED.

**Fig. 1:**
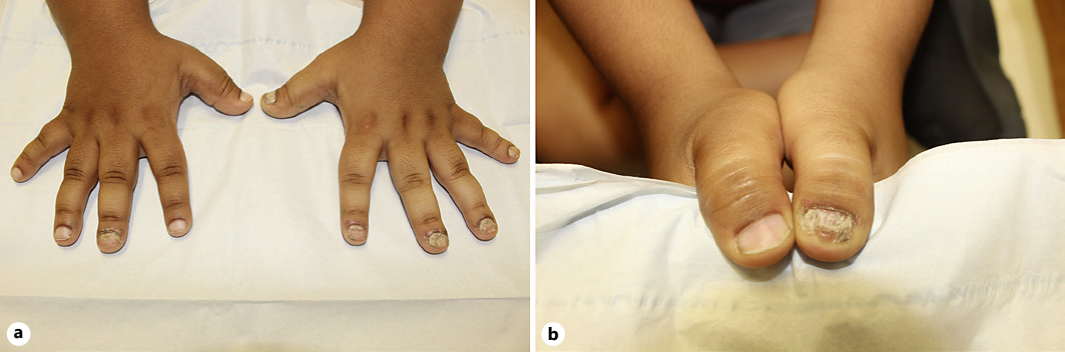
**a, b**. Physical examination of the fingernails showed longitudinal ridges and grooves, thinning, and distal splitting in a child with multiple epiphyseal dysplasia.

**Fig. 2:**
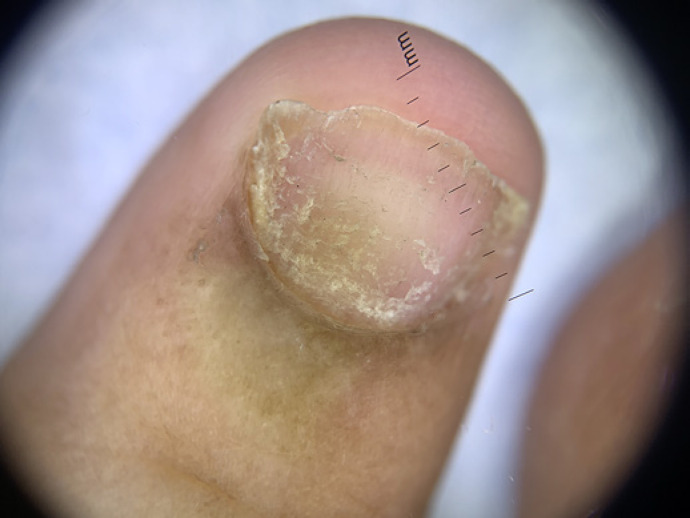
Dermoscopy showing superficial desquamation.

**Fig. 3:**
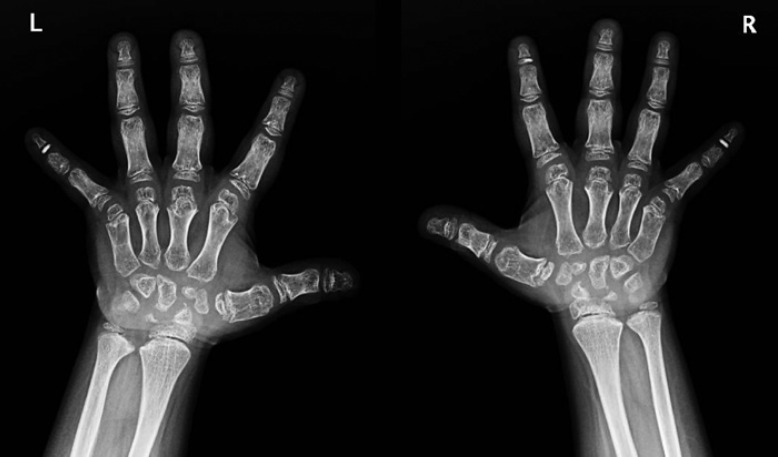
X-ray of hands showing bilateral brachydactyly with shortening of the metacarpals, as well as osseous structures that are diffusely abnormal and irregular in appearance.

On physical examination, fingernails showed longitudinal ridges and grooves, thinning, and distal splitting (Fig. 1a, b). Dermoscopy revealed superficial desquamation (Fig. 2). Nail clippings showed onychodystrophy with entrapment of serum, blood, and leukocytes in the absence of any discernible microbial pathogens. There was no evidence of changes indicative of psoriasis or fungal elements. X-rays of the hands showed osseous structures that were diffusely abnormal and irregular in appearance (Fig. 3). There was brachydactyly with shortening of the metacarpals. The epiphyses of the bilateral 5th distal phalanges and the right 2nd distal phalanx were sclerotic, which is characteristic of a skeletal dysplasia. There was no evidence of an inflammatory or erosive arthritis, and no acute fracture or dislocation.

The patient was prescribed clobetasol 0.05% ointment nightly for 3 weeks on and 1 week off for 3 months with much improvement. He was also counseled to avoid picking to prevent koebnerization.

## Conclusion

This is the first reported case of onychodystrophy in association with MED. Several known skeletal disorders have associated nail changes (Table 1). Hutchinson-Gilford Progeria syndrome is a skeletal dysplasia characterized by dysmorphic facies, short stature, decreased joint mobility, camptodactyly, and nail dystrophy [5]. The nails are short and start off thin but later become thickened and difficult to cut [6]. Coffin-Siris Syndrome is a disorder in which patients present with growth retardation, microcephaly, coarse facies, sparse scalp hair, lax joints, and anonychia or nail hypoplasia, usually in the fifth fingers and toes [7]. Nail-Patella syndrome is due to mutations in the LMX1B gene, encoding a LIM homeodomain transcription factor regulating collagen IV expression and limb dorsoventral formation. These patients present with a small or absent patella and dysplastic nails or anonychia [7, 8]. Congenital brachydactyly is defined as finger or toe shortening. In 1951, several cases of brachydactyly were described in association with anonychia or nail hypoplasia [6]. In 1968, a syndrome of familial nail dysplasia and brachydactyly due to absent middle phalanges of the fingers and toes was documented [7]. In 1986, there was a case report of an 82-year-old woman with brachydactyly of the 3rd and 4th fingers with nail hypoplasia [9].

It is probable that the nail changes in these patients are intimately linked to their skeletal deformities because normal nail development is dependent on appropriate phalangeal formation. Mesenchymal condensation that forms the distal phalanx simultaneously occurs during epidermal thickening and nail fold formation, which requires expression of genes Bmp4, noggin, and Wnt-7a [10, 11]. In a subsequent phase of nail development, MSX1 and MSX2 are involved in appendage growth; MSX1 is associated with proliferation of the germinal matrix of the nail and MSX2 results in differentiation of matrix cells and induction of the nail bed and plate [10, 11]. Nail size is determined by the shape of the distal phalanx, and nail growth rate is associated with the distal phalanx size [10, 11]. This patient with MED and onychodystrophy had nails that appeared similar to nails seen in Hutchinson-Gilford Progeria syndrome, in that they were thickened, as opposed to hypoplastic nails seen in brachydactyly or Coffin-Siris syndrome.

In sum, our case demonstrates that there is a significant association between MED and nail disease, supporting the link between phalangeal formation and nail development. Therefore, it is important to perform a careful examination of the nail units in patients with skeletal dysplasia and screen patients with characteristic and unexplained nail changes for bony changes. Living with skeletal disease is extremely challenging, and treatment of associated nail disease can improve quality of life for these patients.

## Statement of Ethics

Written informed consent was obtained from the patient's parents for publication of details of their medical case and the photographic images. As this is a case report, ethical approval is not required for this study in accordance with the Weill Cornell Institutional Review Board.

## Conflict of Interest Statement

Ms. Albucker has no conflicts of interest. Dr. Lipner has served as a consultant for Hoth Therapeutics, Ortho-Dermatologics, and BelleTorus Corporation.

## Funding Sources

The authors did not receive any funding to complete this research.

## Author Contributions

Ms. Albucker and Dr. Lipner both contributed to the conception, drafting, and revising of the work, as well as final approval of the version to be published. Both Ms. Albucker and Dr. Lipner agree to be accountable for all parts of the work, ensuring that questions related to the accuracy or integrity of any part of the work are appropriately investigated and resolved.

## Data Availability Statement

All data generated or analyzed during this study are included in this article. Further inquiries can be directed to the corresponding author.

## Figures and Tables

**Table 1:** Onychodystrophy in skeletal disorders

Syndrome	Clinical presentation	Ref.
Hutchinson-Gilford Progeria syndrome	Dysmorphic facies, short stature, decreased joint mobility, camptodactyly, nails that appear short and begin as thin nails but later become thickened	5, 6

Coffin-Siris syndrome	Growth retardation, microcephaly, coarse facies, sparse scalp hair and lax joints, anonychia or nail hypoplasia usually in the fifth fingers and toes	7

Nail-Patella syndrome	Small or absent patella, dysplastic nails, or anonychia	8

Brachydactyly Type B	Hypoplastic or absent distal phalanges of fingers, fingernail hypoplasia	10

Brachydactyly Type A5	Absent middle and distal phalanges of hands and feet, fingernail dysplasia	10

Cooks Syndrome	Hypoplastic or absent digits or hand and feet, hypoplastic nails, anonychia, toenail aplasia	10

Ellis-van Creveld syndrome	Disproportionate dwarfism, thoracic dysplasia, congenital heart diseases, genitourinary anomalies, postaxial polydactyl brachydactyly, nail dysplasia	12

Weyers acrofacial dysostosis	Dental anomalies, postaxial polydactyl, mild short stature, nail dystrophy	12
